# Evaluating SARS-CoV-2 antibody reactivity to natural exposure and inactivated vaccination with peptide microarrays

**DOI:** 10.3389/fimmu.2023.1079960

**Published:** 2023-02-20

**Authors:** Peiyan Zheng, Jing Ma, Jiao Yang, Baolin Liao, Zhangkai J. Cheng, Mingshan Xue, Shiyun Li, Yanting Fang, Runpei Lin, Guizhen Zhang, Huimin Huang, Fengyu Hu, Hongwei Ma, Baoqing Sun

**Affiliations:** ^1^ Department of Allergy and Clinical Immunology, Guangzhou Institute of Respiratory Health, State Key Laboratory of Respiratory Disease, National Center for Respiratory Medicine, Guangzhou Laboratory, The First Affiliated Hospital of Guangzhou Medical University, Guangzhou, China; ^2^ Department of Laboratory, Guangzhou Institute of Respiratory Health, State Key Laboratory of Respiratory Disease, National Center for Respiratory Medicine, Guangzhou Laboratory, The First Affiliated Hospital of Guangzhou Medical University, Guangzhou, China; ^3^ Department of Clinical Laboratory, Luoyang Central Hospital Affiliated to Zhengzhou University, Henan, China; ^4^ Division of Nanobiomedicine, Suzhou Institute of Nano-Tech and Nano-Bionics, Chinese Academy of Sciences, Suzhou, China; ^5^ Guangzhou Eighth People’s Hospital, Guangzhou Medical University, Guangzhou, China

**Keywords:** SARS-CoV-2, asymptomatic infections, antibody response, peptide microarrays, vaccination

## Abstract

**Objective:**

Vaccination is effective tool for preventing and controlling SARS-CoV-2 infections, and inactivated vaccines are the most widely used type of vaccine. In order to identify antibody-binding peptide epitopes that can distinguish between individuals who have been vaccinated and those who have been infected, this study aimed to compare the immune responses of vaccinated and infected individuals.

**Methods:**

SARS-CoV-2 peptide microarrays were used to assess the differences between 44 volunteers inoculated with the inactivated virus vaccine BBIBP-CorV and 61 patients who were infected with SARS-CoV-2. Clustered heatmaps were used to identify differences between the two groups in antibody responses to peptides such as M1, N24, S15, S64, S82, S104, and S115. Receiver operating characteristic curve analysis was used to determine whether a combined diagnosis with S15, S64, and S104 could effectively distinguish infected patients from vaccinated individuals.

**Results:**

Our findings showed that the specific antibody responses against S15, S64, and S104 peptides were stronger in vaccinators than in infected persons, while responses to M1, N24, S82, and S115 were weaker in asymptomatic patients than in symptomatic patients. Additionally, two peptides (N24 and S115) were found to correlate with the levels of neutralizing antibodies.

**Conclusion:**

Our results suggest that antibody profiles specific to SARS-CoV-2 can be used to distinguish between vaccinated individuals and those who are infected. The combined diagnosis with S15, S64, and S104 was found to be more effective in distinguishing infected patients from those who have been vaccinated than the diagnosis using individual peptides. Moreover, the specific antibody responses against the N24 and S115 peptides were found to be consistent with the changing trend of neutralizing antibodies.

## 1 Introduction

The Coronavirus Disease 2019 (COVID-19) pandemic, which started at the end of December 2019, has caused tremendous damage to global health and economic development. It is an infectious respiratory disease caused by the severe acute respiratory syndrome coronavirus 2 (SARS-CoV-2), a single-stranded positive-sense RNA virus that is highly unstable and prone to mutation (1).

The mutation rate of SARS-CoV-2 is estimated to be 8 x 10-4/site/year, 1/6 to 1/21 of the mutation rate of influenza viruses (2, 3). This has led to the emergence of several variants of the virus, such as the Alpha (B.1.1.7), Beta (B.1.351), Gamma (P.1), Delta (B.1.617.2), and Omicron (B.1.1.529) variants (4). In light of this, understanding the specificity of immunity against SARS-CoV-2 proteins is of increasing importance in order to determine how antibody response proteins change in humans and the effect on natural immunity and vaccine-induced immunity (5).

To this end, conventional antibody detection methods such as serological detection and indirect immunofluorescence have been adapted and supplemented by the use of protein microarrays (6). In such microarrays, small molecules like polypeptides and proteins are immobilized on microfabricated surfaces to enable high-throughput screening studies (7). These proteinchips are useful for screening unknown antibodies to certain antigens, using the affinity of components to certain proteins as an indicator (8). A SARS-CoV-2 proteome chip has been developed to provide an effective tool for the diagnosis of COVID-19, with high accuracy, low sample consumption, and a simple and rapid operation (9).

In the past decade, there has been an alarming increase in the number of outbreaks caused by severe acute respiratory syndrome (SARS), Ebola, and coronavirus variants, highlighting the importance of rapid disease diagnosis and vaccine development (10). To this end, the identification of biomarkers and the development of antigenic targets for vaccines has become of paramount importance. Peptide microarrays are a powerful tool in this regard, as they can display numerous putative target proteins that can be rapidly translated into overlapping linear (and cyclic) peptides for multiplexed high-throughput antibody analysis (11).

The coronavirus spike (S) protein is a characteristic structural component of the viral envelope and has been identified as a key target for vaccines to prevent infection (12). Several experiments have been conducted to date on constructing SARS-CoV-2 antigen microarrays as diagnostic tools, analyzing peptides to predict vaccination efficacy (13), and accurately assessing the impact of COVID-19 in epidemics (14). Differentiation and diagnosis are essential for vaccine development. For this purpose, in this study, we conducted microarray screening for the full-length SARS-CoV-2 proteins in patients and healthy cohorts, and identified 11 peptides with a high response in patients (M1, N16, N24, S15, S39, S44, S64, S82, S95, S104, and S115). SARS-CoV-2-specific antibody profiles can reliably distinguish COVID-19 patients from vaccinated and asymptomatic individuals, providing a valuable tool for large-scale population surveillance studies to accurately estimate the true prevalence of the disease.

## 2 Materials and methods

### 2.1 Ethics approval

This study was conducted in accordance with the ethical principles of the Declaration of Helsinki and was approved by the Medical Ethics Committee of the First Affiliated Hospital of Guangzhou Medical University (ethics approval no. gyfyy-2021-31) and Guangzhou Eighth People’s Hospital (202002135). Written informed consent was obtained from all participants prior to the study, in strict adherence to the ethical standards outlined in the Declaration of Helsinki.

### 2.2 Volunteers and patients characteristics and study design

Forty-four healthy adult volunteers (n = 44) were randomly selected for follow-up visits before, one month after each of the three doses of the inactivated COVID-19 vaccination BBIBP-CorV, and were randomly selected for follow-up visits before immunization (before the first dose, i.e., healthy subjects, V1), one month after the first injection (V1+30), one month after the second injection (V2+30), and one month after the booster injection (V3+30). Out of these 44 volunteers, 22 had a hypoimmune response, indicating that no effective neutralizing antibody (NAb) was produced one month after the second dose, defined as virus neutralization test (VNT) <8, while the remaining 22 volunteers exhibited a hyperimmune response (VNT ≥16).

Furthermore, 61 patients with SARS-CoV-2 infection, confirmed using real-time quantitative polymerase chain reaction (RT-qPCR) and hospitalized in Guangzhou Eighth People’s Hospital, were divided into three groups according to the severity of their disease: 18 asymptomatic patients (AP), 33 mildly-symptomatic patients (MP), and 10 severely-symptomatic patients (SP). As asymptomatic infections do not develop symptoms, the day on which the nucleic acid test was first positive was defined as day 0.

### 2.3 Blood collection and preparation

Peripheral blood samples were collected from the antecubital vein after an overnight fast in 10 ml EDTA and 5 ml Serum-Gel tubes. The tubes were immediately centrifuged at 3000 rpm at 4°C for 10 minutes to separate the plasma and serum, respectively. Following centrifugation, all samples were aliquoted into 0.5 ml tubes and stored at -80°C until further processing. Plasma was used for live-virus neutralization assay and serum was used for antibodies and peptide microarrays detection.

### 2.4 RT-PCR-based detection and SARS-CoV-2 infection determination

Nucleic acids were extracted from samples primarily collected from nasopharyngeal tissue. The extraction was conducted according to the instructions of a commercial viral RNA extraction kit (DaAn Gene Co., Ltd., Sun Yat-sen University, China). Subsequently, reverse transcription-polymerase chain reaction (RT-PCR) assay kits targeting the SARS-CoV-2 open reading frame1ab (ORFlab) and nucleocapsid (N) gene regions were acquired from DaAn Gene Co., Ltd. (Guangzhou, China). All extraction and testing processes were conducted in accordance with scientific reporting standards.

### 2.5 Peptides

The amino acid sequence of the SARS-CoV-2 strain (MN908947) was analyzed and the 20-mer peptides with an overlap of 10 amino acid (aa) residues, partially covering four structural proteins of SARS-CoV-2 (i.e., Spike, Envelope, Membrane, and N proteins), were chemically synthesized by GenScript (Jiangsu, China). The microarray yielded 131 peptides (**Table S1**). In order to assess the protein-peptide interactions, a peptide and protein hybrid microarray (PPHM) was designed using RBD (GenScript, Jiangsu, China), (S1+S2) ECD (Sino Biological, Beijing, China) and the Nucleotide protein (N protein, VACURE Biotechnology, Sichuan, China) and 131 peptides of SARS-CoV-2, which finally yielded 136 peptides and proteins in total. Each well of the chip had a 4x4 rectangular microarray, with three human immunoglobulin G (IgG)-positive controls and one negative control in the four corners. The PPHM was screened to obtain the indicated peptides for detecting COVID-19 patients with both high sensitivity and high specificity, detailed descriptions was showed in supplementary material (Appendix 1). The probes included in the center of the microarray were M1, N16, N24, S15, S39, S44, S64, S82, S95, S104, and S115 (see **Table 1**).

**Table 1:** Peptide sequences.

Peptide	Position (base-pairs)	Sequence
S15	141–160	LGVYYHKNNKSWMESEFRVY
S39	381–400	GVSPTKLNDLCFTNVYADSF
S44	431–450	GCVIAWNSNNLDSKVGGNYN
S64	631–650	PTWRVYSTGSNVFQTRAGCL
S82	811–830	KPSKRSFIEDLLFNKVTLAD
S95	941–960	TASALGKLQDVVNQNAQALN
S104	1031–1050	ECVLGQSKRVDFCGKGYHLM
S115	1141–1160	LQPELDSFKEELDKYFKNHT
M1	1–20	MADSNGTITVEELKKLLEQW
N16	151–170	PANNAAIVLQLPQGTTLPKG
N24	231–250	ESKMSGKGQQQQGQTVTKKS

### 2.6 Detection of peptide binding antibodies in serum by microarray

The screening process was conducted following the same procedure as described previously (15) with minor modifications and using the indirect enzyme-linked immunosorbent assay (indirect ELISA) principle. To begin, diluted serum (100-fold) was prepared using a serum-dilution buffer containing 1% bovine serum albumin, 1% casein, 0.5% sucrose, 0.2% polyvinylpyrrolidone, 0.5% Tween 20 in 0.01 M phosphate-buffered saline (PBS, pH 7.4). A 100 μL sample of the diluted serum was then added to each microarray well and incubated with a peptide microarray for 30 minutes on a shaker (500 rpm, 37°C). The microarray well incubated with just serum-dilution buffer served as a negative control. Subsequently, the microarray was washed three times with 0.01 MPBS-Tween (PBST, pH 7.4) and then incubated with 100 μL of horseradish peroxidase (HRP)-conjugated anti-human IgG (ZSGB-BIO, Beijing, China) for a further 30 minutes on a shaker (500 rpm, 37°C). Following this, any unbound HRP-conjugated anti-human IgG was washed away with PBST and 100 μL of 1-step Ultra TMB-Blotting Solution (Thermo Scientific) was used to detect any informative signal of IgGs against peptide probes using a microarray imager (Suzhou Epitope, China). The data were then processed using UVC instrument V1.0 software (version Epitope Company, Suzhou, China). The signal for each dot was calculated by subtracting the background signal from the readout signal: Signal _dot_ = Signal _readout_ - Signal _background_. The cut-off value for each probe was set at 10.

### 2.7 Focus reduction neutralization test

The FRNT was conducted in a certified biosafety laboratory for live-virus neutralization assay. Briefly, plasma samples were continuously diluted and mixed with 50 µL SARS-CoV-2 virus suspension (100 virus focal forming units, FFU) in a 96-well plate, followed by incubation at 37°C for 1 hour. The mixture was then transferred to a 96-well plate inoculated with Vero E6 cells (ATCC, Manassas, VA) and incubated for an additional hour at 37°C to allow for viral entry into the cells. Subsequently, the cell culture medium was removed and replaced with a covering medium (125 ml 1.6% carboxymethyl cellulose, CMC). The plates were then incubated at 37°C for 24 hours. After removal of the covering, the cells were fixed with 4% paraformaldehyde solution for 30 minutes. Subsequently, cells were permeabilized with 0.2% Triton X-100 and incubated with cross-reactive rabbit Anti-SARS-CoV-N IgG 40143R001 at 37°C for 1 hour, followed by incubation with HRP-conjugated goat anti-rabbit IgG (high+low) antibody (diluted at 1:4,000) (Catalog number:111-035-144, Jackson ImmunoResearch, West Grove, PA). After an additional incubation at 37°C for 1 hour, KPL TrueBlue peroxidase substrate (Seracare Life Sciences Inc., Milford, MA, USA) was used as catalyst for the reaction. Finally, an Elispot reader (Cellular Technology Ltd., Shaker Heights, OH) was employed to calculate the number of SARS-CoV-2 lesions.

### 2.8 Statistical analysis

Statistical analyses were conducted with SPSS software (version 25.0). The Mann-Whitney U test was used to evaluate the measurement data between the two groups, which are described as median and quartile distances. Spearman’s correlation analysis was used to analyze the correlations between the two groups. Furthermore, the ROC curve and AUC analysis were conducted using R pROC package, while accuracy, sensitivity, specificity, and cutoff value were calculated with R caret and epiR packages. In addition, odds ratio and corresponding 95% confidence intervals (CI) from logistic regression (LR) were calculated using the R-package stats. Finally, Excel and Graph Pad Prism 8.0 (La Jolla, USA) were used for charting. Statistical significance was denoted as follows: * *P* < 0.05, ** *P* < 0.01, *** *P* < 0.001, and **** *P* < 0.0001.

## 3 Results

### 3.1 Baseline data

**Figure 1:**
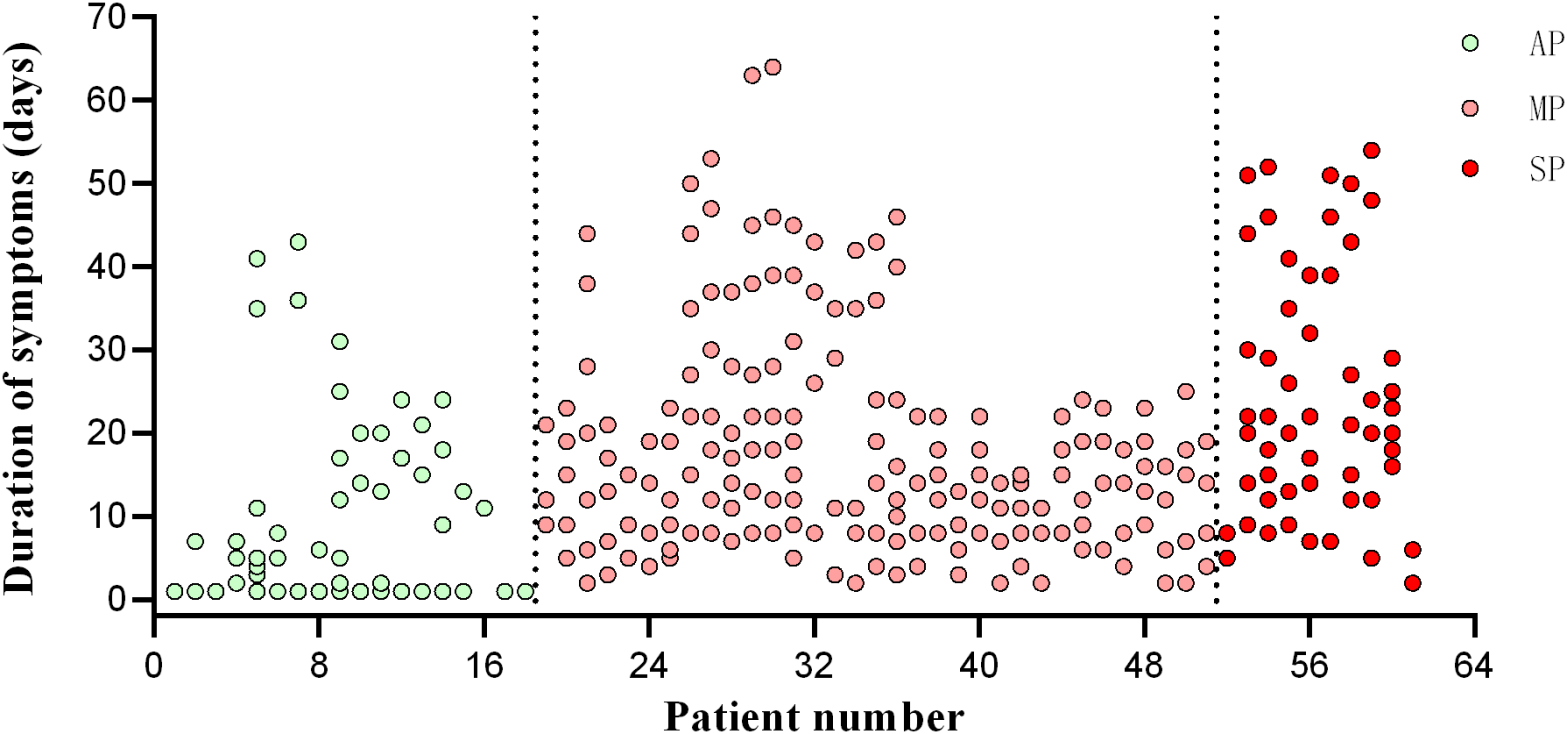
Timepoint of patient follow-up. The abscissa is the patient’s enrolment number, and the ordinate is the day of onset. AP, asymptomatic patients; MP, mildly patients; SP, severely patients.

Forty-four healthy volunteers were enrolled in the study and administered three doses of the BBIBP-CorV vaccine. Follow-up was conducted approximately one month after each dose (**Table 2**). The volunteers were separated into two groups: a hyperimmune response group (n=22), with an average age of 36.14 ± 9.14 years, and a hypoimmune response group (n=22), with an average age of 41.32 ± 5.96 years. **Figure 1** illustrates the corrected sample sizes while the patients returned for follow-up visits.

**Table 2:** Patients and vaccinated baseline data.

Goup		Infections				Vaccinated	
		Asymptomatic	Mildly	Severely		Hyperimmune response	Hypoimmune response
Age (mean ± SD)		31.8 ± 15.5	47.3 ± 14.6	58.8 ± 12.4		36.1 ± 9.1	41.3 ± 6. 0
Gender (Female/Male)		7/11	17/16	9/1		7/15	1/21
Time point of return visit	Week 1	28	31	6	V0	22	22
	Week 2	8	55	10	V1+30	22	22
	Week 3	7	35	11	V2+30	22	22
	Week 4	3	23	8	V3+30	22	22
	Week 5	2	6	5			
	Week 6	2	10	3			
	Week 7–10	0	13	10			
Outcome	Discharge	18	33	10			
	Death	0	0	0			
Highest body temperature	Median ± IQR	36.9 ± 0.2	38.0 ± 0.7	38.2 ± 0.3^*^			
Virus shedding time (days)	Median ± IQR	3.0 ± 4.5	19.0 ± 15.5^*^	19.6 ± 28.4^*^			
Duration of hospitalization (days)	Median ± IQR	8.0 ± 5.0	21.0 ± 5.0^*^	23.0 ± 15.0^*^			

SD, standard deviation; IQR, interquartile range; *P < 0.05 (vs. asymptomatic patients, Mann-Whitney test).

### 3.2 Peptide-specific response differences between vaccinated with hyperimmune and hypoimmune responses

**Figure 2:**
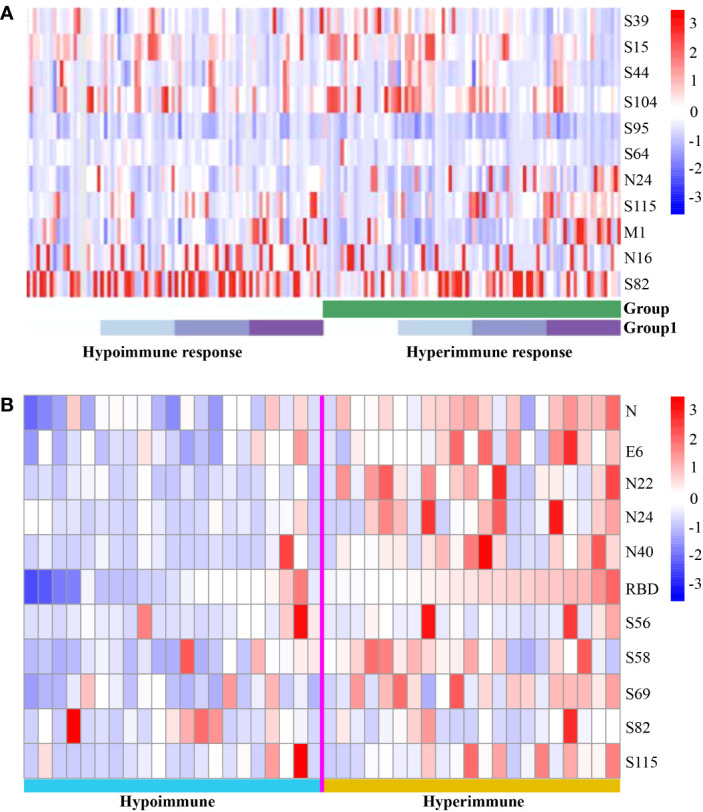
Heatmaps of peptide-specific responses in vaccinated with hypo- and hyperimmune responses. **(A)** Vaccinated were divided into two groups: hypoimmune and hyperimmune (green) responses. Regarding Group 1, light to dark (purple) coloration represents the timepoints before immunization, 30 days after the first dose, 30 days after the second dose, and 30 days after the third dose. **(B)** Peptide reactions collected by V3 + 30 (30 days after the third dose).

We conducted an analysis of the peptide-specific antibody responses in 22 hyperimmune and 22 hypoimmune individuals, as indicated by clustered heatmaps (**Figure 2**). We found that the distribution of the 11 peptides was consistent between the two groups, with no statistically significant difference before the third immunization (**Figure S1**). Our findings suggest that healthy individuals vaccinated with inactivated COVID-19 vaccines produce similar specific antibody response spectra. However, there was a significant variability in the peptide reactions collected by V3+30, and the hypoimmune group showed significantly lower binding to peptides N22, N24, N40, and S58 and S69 compared to the hyperimmune group, while S82 was the opposite. Therefore, the detection method using short peptide antibodies can effectively distinguish between hypoimmune and hyperimmune vaccinated individuals. As such, antibody testing for COVID-19 peptides may be used as a screening tool for hypoimmune populations, helping to alert them to the need to bolster their immunity.

### 3.3 Differences in peptide specific responses between vaccinated and infected individuals

**Figure 3:**
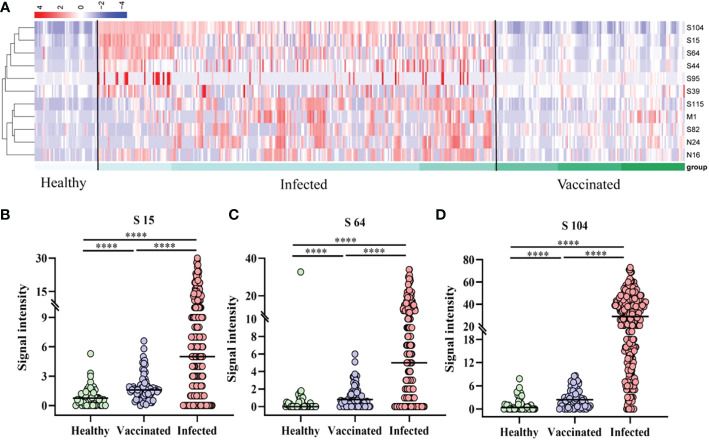
Peptide-specific IgG responses of vaccinated and patients. **(A)** Heatmap of the distribution of peptide-specific reactions between infected individuals and vaccinated. **(B–D)** Specific responses to selected peptides in healthy populations, vaccinated, and patients. **(B)** S15; **(C)** S64; **(D)** S104. ****P ≤ 0.0001.

A cluster heatmap (Fig 3A was used to illustrate the distribution of peptide-specific responses in 44 vaccinated (V1, V1+30, V2+30, and V3+30) and 61 patients (AP, MP, and SP). Analysis revealed that specific IgG responses to the 11 epitopes were significantly weaker in all vaccinated populations than those in all types of patients. Subsequent analysis of S64, S15, and S104 peptides showed statistically significant differences in the specific IgG responses among healthy persons, infected persons, and vaccinees (**Figures 3B–D**).

### 3.4 Differences of peptide-specific responses among AP, MP, and SP

**Figure 4:**
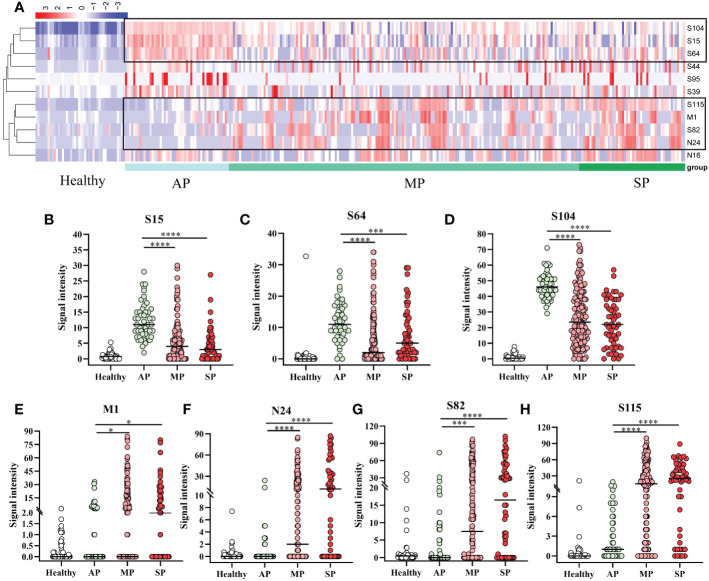
Specific IgG responses to selected peptides in asymptomatic, mild, and severe patients. **(A)** Heatmap of peptide-specific reactions between infected and healthy individuals. **(B)** S15; **(C)** S64; **(D)** S104; **(E)** M1; **(F)** N24; **(G)** S82; **(H)** S115. Healthy, before immunization; AP, asymptomatic patients; MP, mildly patients; SP, severely patients. *P ≤ 0.05; ***P ≤ 0.001; ****P ≤ 0.0001.

As shown in **Figure 3A**, the specific reactivity of peptides differed between vaccinated and patients, and among patients of different affliction grades. To further investigate this, 18 AP, 33 MP, and 10 SP patients were enrolled and subjected to cluster analysis. Results revealed that the specific reactivity of S64, S15, and S104 peptides in AP was significantly higher than in MP and SP (*P* ≤ 0.001). However, there was no statistical difference between MP and SP (**Figures 4B–D**). On the other hand, the expression of M1, N24, S82, and S115 peptides in AP was significantly lower than in MP and SP (*P ≤* 0.05), with no significant difference between MP and SP observed (**Figures 4E–H**).

### 3.5 SARS-CoV-2 peptides associated with neutralizing activity against viruses

**Figure 5:**
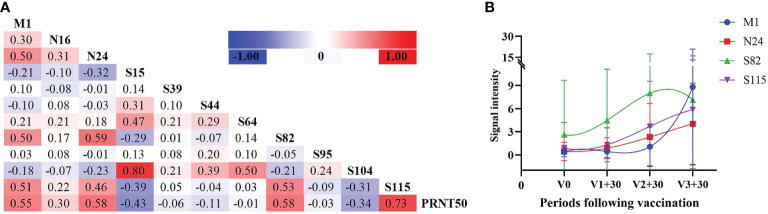
Neutralizing antibody reactions with peptide-specific IgG. **(A)** Correlation analysis between peptides and neutralizing antibodies in infected individuals. **(B)** Peptide-specific IgG response at different time periods following vaccination, selecting four peptides with the highest correlation coefficient against PRNT50.

Screening the antibody-binding activity of the SARS-COV-2 antigen allows for the exploration of existing potential epitopes and characteristics of the infection mechanism, providing a reference for COVID-19 treatment and peptide vaccine development. Therefore, we analyzed the correlation between the SARS-COV-2 peptide-specific IgG response and PRNT50 during infection. According to the correlation heatmap, peptide-specific IgG of M1, N24, S82 and S115 displayed a significant correlation with PRNT50 (0.55, 0.58, 0.58 and 0.73, respectively) (*P* ≤ 0.05, **Figure 5A**). Multiple linear regression analysis confirmed the significance of the correlation with F = 47.758, *P* ≤ 0.001, and R = 0.649. Moreover, the effects of N24 and S115 peptides included in the model on the PRNT50 results were also found to be statistically significant (*P* ≤ 0.05).

It has been observed that antibody levels in vaccinated individuals typically peak one month after vaccination. Therefore, we analyzed the antibody levels of these four peptides one month after the first, second , and third doses included in the study. Additionally, we analyzed the variation trend of these four peptides in vaccinated individuals and found that N24 and S115 were most consistent with the variation trend of antibody levels (**Figure 5B**).

### 3.6 Identification can distinguish the combination of peptides in infected persons from vaccinated persons

With the recent rise in screening efforts in China, an increasing number of patients have been identified as nucleic acid-positive. However, the viral load can reach undetectable levels within 1–2 weeks of symptom onset, making the diagnosis of AP essential for effective epidemic control. Unfortunately, antigen detection is currently plagued by low sensitivity and specificity. Identifying differences in peptide-specific IgG antibody responses between vaccinated, AP, and symptomatic patients (MP and SP) is thus of great importance for disease discovery, diagnosis, and treatment.

**Figure 6:**
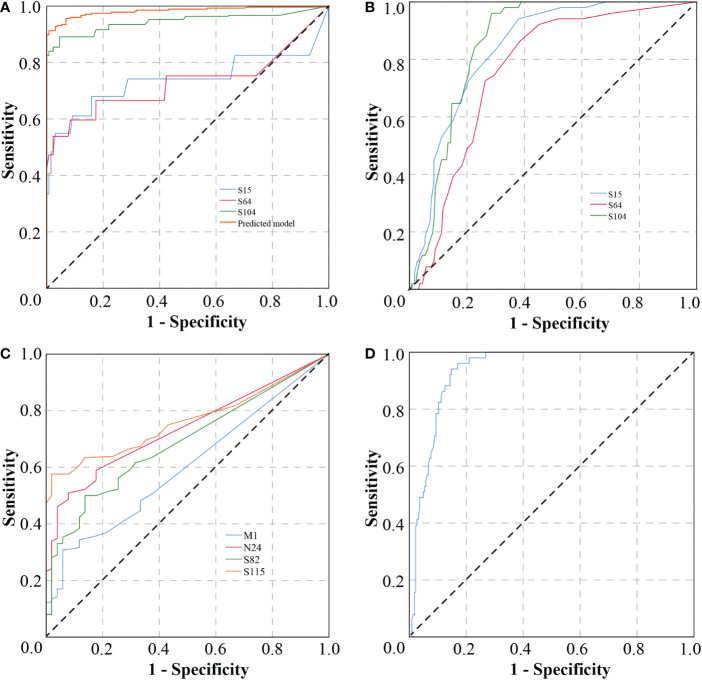
Subject operating curve. **(A)** ROC curves of infected persons vs. vaccinated, with different curves showing predictions using prediction model, peptides S15, S64, and S104. **(B)** ROC curves of asymptomatic and highly-expressed-symptomatic patients, with different curves showing predictions using single peptides. **(C)** ROC curves of asymptomatic and low-expressed-symptomatic patients, with different curves showing predictions using single peptides. **(D)** ROC curve of asymptomatic and symptomatic patients, predicted using the prediction model.

To this end, we used three markers, S64, S15, and S104, to conduct ROC analysis on patients after vaccination and on infected patients (**Figure 6A**). We found that these markers had both good sensitivity and specificity for distinguishing between patients and vaccinees, with combined diagnosis achieving a sensitivity of 91.3% and a specificity of 99.2% (**Table 3**).

**Table 3:** Receiver operating characteristic (ROC) curve parameters between infections and vaccinated.

Peptide	Area	95% confidence interval (CI)		Sensitivity	Specificity	Cutoff
		Lower bound	Upper bound			
S15	0.739	0.692	0.787	0.68	0.841	2.95
S64	0.73	0.682	0.777	0.538	0.977	3.85
S104	0.946	0.923	0.968	0.891	0.955	6.95
Combined diagnosis	0.982	0.971	0.994	0.913	0.992	–

In addition, to differentiate AP from symptomatic patients, ROC analysis was conducted for S64, S15, and S104 with high expression in AP (**Figure 6B**) and M1, N24, S82, and S115 with low expression (**Figure 6C**). The sensitivities of S64, S15, and S104 to distinguish AP from symptomatic patients were 94.1%, 86.3%, and 96.1%, respectively, while the specificities of M1, N24, S82, and S115 were 94.1%, 92.2%, 86.3%, and 98%, respectively (**Table 4**). The combined predictive sensitivity and specificity of the diagnostics reached 94.1% and 85.3%, respectively (**Figure 6D**; **Table 4**), making them suitable biomarkers to distinguish AP from symptomatic patients.

**Table 4:** Receiver operating characteristic (ROC) curve parameters between symptomatic and asymptomatic patients.

Peptide	Area	95% confidence interval (CI)		Sensitivity	Specificity	Cutoff
		Lower bound	Upper bound			
S15	0.84	0.79	0.89	0.941	0.621	5.5
S64	0.761	0.698	0.824	0.863	0.616	5.5
S104	0.856	0.812	0.899	0.961	0.714	34.5
M1	0.601	0.524	0.679	0.308	0.941	11.5
N24	0.736	0.673	0.798	0.509	0.922	3.5
S82	0.687	617	0.757	0.5	0.863	10.5
S115	0.763	0.707	0.818	0.576	0.98	12.5
Combined diagnosis	0.935	0.907	0.963	0.941	0.853	–

## 4 Discussion

Herd immunity achieved through vaccination is the best way to combat the COVID-19 pandemic (1). To study SARS-CoV-2, scientists developed various antibody detection methods through serological research. This included analyzing factors such as pathogenesis, transmission rate, and infection efficacy, as well as studying hyperimmune and hypoimmune responses. Sera from individuals with positive live virus antibody detection were used to detect SARS-CoV-2 short peptides and observe specific antibody responses, which allowed us to distinguish between distinct groups (16). According to the heatmap results, the response intensity of the vaccinated population to the short peptide at the corresponding site was significantly weaker than that in all types of patients. Compared to individuals infected with SARS-CoV-2, the antibody responses to the S and N proteins of the inactivated vaccines were significantly weakened (13), which indicated that those vaccinated with the inactivated virus vaccine produced lower antibody responses than those who were actually infected.

Our experiments showed that the antibody response of vaccinated populations to the short peptides was significantly weaker than that of infected individuals. In addition, S15, S64, and S104 levels were low in vaccinated patients and highest in the AP group. ROC curves for these peptides indicated low sensitivity but high specificity and no high diagnostic value for S15 and S64. However, the combined diagnosis of S15, S64 and S104 had a sensitivity and specificity of 0.913 and 0.992, respectively, and could thus be used to distinguish vaccinated individuals from patients.

Studies conducted on COVID-19 protein microarrays to determine biomarkers that can differentiate between vaccinees and COVID-19 patients reached similar conclusions (8, 13, 17–19). For example, Ma et al. (2021) found that S, N, and NSP7 proteins can be used to distinguish inactivated vaccine recipients from COVID-19 patients (13). Our experiments not only differentiated vaccine recipients from patients, but also distinguished between AP, MP, and SP. We observed that the S protein, RBD, and two polypeptides (S1-5 and S2-22) can be used to evaluate the protective effect of inactivated vaccines and are potential markers for SARS-CoV-2-specific immune evaluation. Nucleocapsid antibodies were found to be biomarkers of natural exposure to SARS-CoV-2, which can be used to distinguish those previously exposed to the virus in vaccinated populations (8).

Further research is needed to determine the optimal combination of antigens for the most accurate detection of specific coronavirus antibodies. In addition, the antibody levels of convalescent patients and the duration of the protective effect of neutralizing antibody levels should be considered to better distinguish between biomarkers of convalescent patients and vaccine recipients. Also, the cross-reactive antibody response of SARS-CoV-1 and other common HCoVs, MERS-CoV, and common cold-causing coronaviruses should be investigated.

Overall, antibody detection assays for SARS-CoV-2 short peptide chips are essential for individual samples recovered from SARS-CoV-2 infection and can reflect herd immunity at the population level. Such an approach can be used to determine individual disease risk and identify new infection waves, becoming a major advantage in vaccine development and vaccine immunogenicity (6)

## Data availability statement

The original contributions presented in the study are included in the article/**Supplementary Material**. Further inquiries can be directed to the corresponding authors.

## Ethics statement

The studies involving human participants were reviewed and approved by the Medical Ethics Committee of the First Affiliated Hospital of Guangzhou Medical University (ethics approval no. gyfyy-2021-31) and Guangzhou Eighth People’s Hospital (202002135). The patients/participants provided their written informed consent to participate in this study. Written informed consent was obtained from the individual(s) for the publication of any potentially identifiable images or data included in this article.

## Author contributions

All authors made a significant contribution to the work reported, whether that is in the conception, study design, execution, acquisition of data, analysis, and interpretation, or in all these areas; took part in drafting, revising, or critically reviewing the article; gave final approval of the version to be published; have agreed on the journal to which the article has been submitted; and agree to be accountable for all aspects of the work.
