# Supplementary figures and images for: Evaluating SARS-CoV-2 antibody reactivity to natural exposure and inactivated vaccination with peptide microarrays
Source: Front Immunol. 2023 Feb 20;14:1079960. doi: 10.3389/fimmu.2023.1079960 (PMC9986310; doi:10.3389/fimmu.2023.1079960)

S1 A

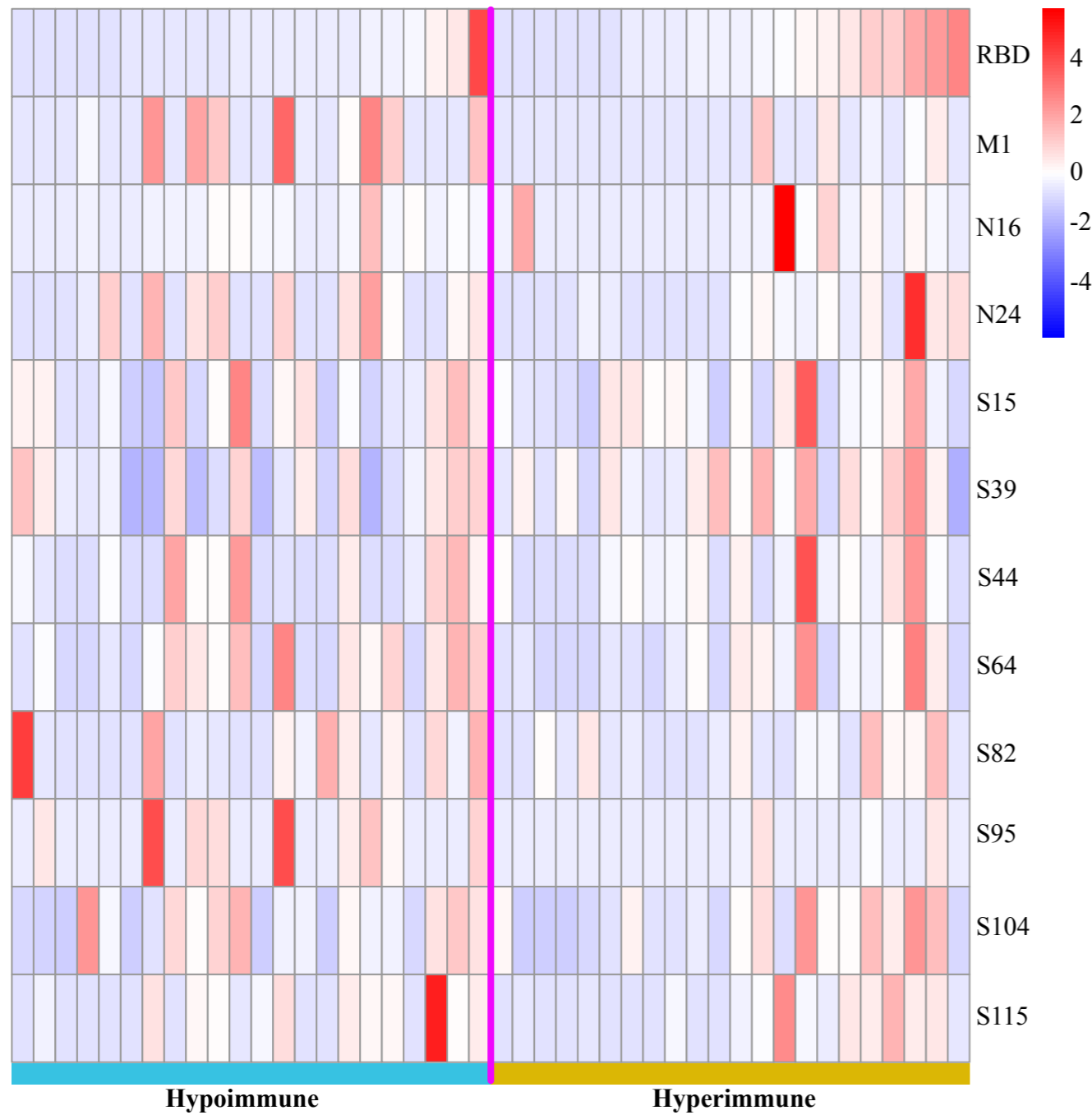

S1 B

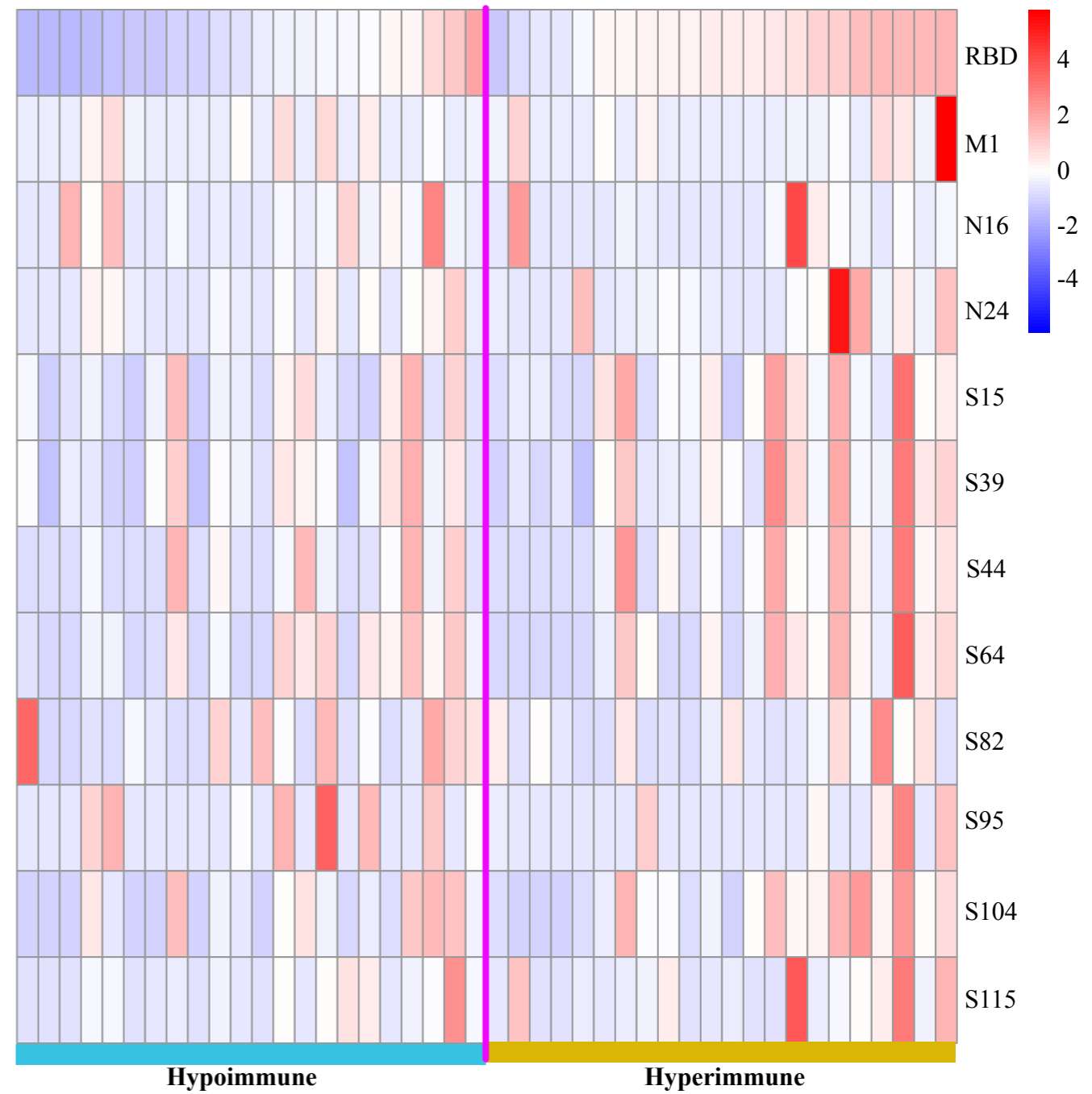

Supplement: Supplementary file 1 [file Image_1.pdf]
