# Supplementary material for: Evaluating SARS-CoV-2 antibody reactivity to natural exposure and inactivated vaccination with peptide microarrays
Source: Front Immunol. 2023 Feb 20;14:1079960. doi: 10.3389/fimmu.2023.1079960 (PMC9986310; doi:10.3389/fimmu.2023.1079960)
Supplement: Supplementary file 2 [file DataSheet_1.docx]

**SUPPLEMENTAL MATERIAL**

**Appendix 1. Indicated peptides selecting**

We selected the indicated peptides in this study mainly based on our previous screening results by using peptide and protein hybrid microarray (PPHM). In brief, we first purchased RBD (GenScript, Jiangsu, China), (S1+S2) ECD (Sino Biological, Beijing, China) and the Nucleotide protein (N protein, VACURE Biotechnology, Sichuan, China) of SARS-CoV-2 and set them as protein probes to fabricate the microarray for COVID-19 serum screening. By analyzing the amino acid sequence of SARS-CoV-2 strain (MN908947), 20-mer peptides with an overlap of 10 aa residues, partially covering four structure proteins (S, N, M and E) of SARS-CoV-2 were chemically synthesized by GenScript (Jiangsu, China), which finally yielded 136 peptides in total. Thus, our PPHM comprises three SARS-CoV-2 related protein probes and 136 derived peptide probes. PPHM was screened against 973 serum samples (500 confirmed COVID-19 serum and 473 negative serum samples) and further validated by using 414 serum samples to obtain the probes for detecting COVID-19 patients with both high sensitivity and high specificity. The serum responses to indicated peptides (as shown in our study) could well discriminate between COVID-19 and control serum samples (P < 0.001), much better than protein probes. To improve the screening efficiency, we directly took the advantage of these peptides in our present study. Peptide derived proteins, such as (S1+S2) ECD and N, or SARS-CoV-2 related protein RBD have already been used for previous screenings, thus we did not enroll them for the present study.
