# Supplementary material for: Evaluating SARS-CoV-2 antibody reactivity to natural exposure and inactivated vaccination with peptide microarrays
Source: Front Immunol. 2023 Feb 20;14:1079960. doi: 10.3389/fimmu.2023.1079960 (PMC9986310; doi:10.3389/fimmu.2023.1079960)
Supplement: Supplementary file 3 [file Table_1.pdf]

Tabel S1. Peptides in Microarray.

PepID	Position	Sequence
P-S2	11-30	VSSQCVNLTTRTQLPPAYTN
P-S3	21-40	RTQLPPAYTNSFTRGVYYPD
P-S4	31-50	SFTRGVYYPDKVFRSSVLHS
P-S5	41-60	KVFRSSVLHSTQDLFLPFFS
P-S6	51-70	TQDLFLPFFSNVTWFHAIHV
P-S7	61-80	NVTWFHAIHVSGTNGTKRFD
P-S8	71-90	SGTNGTKRFDNPVLPFNDGV
P-S10	91-110	YFASTEKSNIIRGWIFGTTL
P-S14	131-150	CEFQFCNDPFLGVYYHKNNK
P-S15	141-160	LGVYYHKNNKSWMESEFRVY
P-S18	171-190	VSQPFLMDLEGKQGNFKNLR
P-S19	181-200	GKQGNFKNLREFVFKNIDGY
P-S20	191-210	EFVFKNIDGYFKIYSKHTPI
P-S21	201-220	FKIYSKHTPINLVRDLPQGF
P-S22	211-230	NLVRDLPQGFSALEPLVDLP
P-S24	231-250	IGINITRFQTLLALHRSYLT
P-S25	241-260	LLALHRSYLTPGDSSSGWTA
P-S27	261-280	GAAAYYVGYLQPRTFLLKYN
P-S29	281-300	ENGTITDAVDCALDPLSETK
P-S31	301-320	CTLKSFTVEKGIYQTSNFRV
P-S32	311-330	GIYQTSNFRVQPTESIVRFP
P-S34	331-350	NITNLCPFGEVFNATRFASV
P-S35	341-360	VFNATRFASVYAWNRKRISN
P-S36	351-370	YAWNRKRISNCVADYSVLYN
P-S37	361-380	CVADYSVLYNSASFSTFKCY
P-S38	371-390	SASFSTFKCYGVSPTKLNDL
P-S39	381-400	GVSPTKLNDLCFTNVYADSF
P-S41	401-420	VIRGDEVRQIAPGQTGKIAD
P-S42	411-430	APGQTGKIADYNYKLPDDFT
P-S44	431-450	GCVIAWNSNNLDSKVGGNYN
P-S45	441-460	LDSKVGGNYNYLYRLFRKSN
P-S46	451-470	YLYRLFRKSNLKPFERDIST
P-S48	471-490	EIYQAGSTPCNGVEGFNCYF
P-S49	481-500	NGVEGFNCYFPLQSYGFQPT
P-S50	491-510	PLQSYGFQPTNGVGYQPYRV
P-S51	501-520	NGVGYQPYRVVVLSFELLHA
P-S52	511-530	VVLSFELLHAPATVCGPKKS
P-S53	521-540	PATVCGPKKSTNLVKNKCVN
P-S54	531-550	TNLVKNKCVNFNFNGLTGTG
P-S55	541-560	FNFNGLTGTGVLTESNKKFL
P-S56	551-570	VLTESNKKFLPFQQFGRDIA
P-S57	561-580	PFQQFGRDIADTTDAVRDPQ
P-S58	571-590	DTTDAVRDPQTLEILDITPC
P-S61	601-620	GTNTSNQVAVLYQDVNCTEV
P-S62	611-630	LYQDVNCTEVPVAIHADQLT
P-S63	621-640	PVAIHADQLTPTWRVYSTGS
P-S64	631-650	PTWRVYSTGSNVFQTRAGCL
P-S66	651-670	IGAEHVNNSYECDIPIGAGI
P-S68	671-690	CASYQTQTNSPRRARSVASQ
P-S69	681-700	PRRARSVASQSIIAYTMSLG
P-S76	751-770	NLLLQYGSFCTQLNRALTGI

P-S78	771-790	AVEQDKNTQEVFAQVKQIYK
P-S79	781-800	VFAQVKQIYKTPPIKDFGGF
P-S80	791-810	TPPIKDFGGFNFSQILPDPS
P-S82	811-830	KPSKRSFIEDLLFNKVTLAD
P-S83	821-840	LLFNKVTLADAGFIKQYGDC
P-S84	831-850	AGFIKQYGDCLGDIAARDLI
P-S85	841-860	LGDIAARDLICAQKFNGLTV
P-S86	851-870	CAQKFNGLTVLPPLLTDEMI
P-S90	891-910	GAALQIPFAMQMAYRFNGIG
P-S91	901-920	QMAYRFNGIGVTQNVLYENQ
P-S92	911-930	VTQNVLYENQKLIANQFNSA
P-S93	921-940	KLIANQFNSAIGKIQDSLSS
P-S94	931-950	IGKIQDSLSSTASALGKLQD
P-S95	941-960	TASALGKLQDVVNQNAQALN
P-S96	951-970	VVNQNAQALNTLVKQLSSNF
P-S98	971-990	GAISSVLNDILSRLDKVEAE
P-S101	1001-1020	LQSLQTYVTQQLIRAAEIRA
P-S102	1011-1030	QLIRAAEIRASANLAATKMS
P-S103	1021-1040	SANLAATKMSECVLGQSKRV
P-S104	1031-1050	ECVLGQSKRVDFCGKGYHLM
P-S105	1041-1060	DFCGKGYHLMSFPQSAPHGV
P-S106	1051-1070	SFPQSAPHGVVFLHVTYVPA
P-S107	1061-1080	VFLHVTYVPAQEKNFTTAPA
P-S108	1071-1090	QEKNFTTAPAICHDGKAHFP
P-S109	1081-1100	ICHDGKAHFPREGVFVSNGT
P-S110	1091-1110	REGVFVSNGTHWFVTQRNFY
P-S111	1101-1120	HWFVTQRNFYEPQIITTDNT
P-S114	1131-1150	GIVNNTVYDPLQPELDSFKE
P-S115	1141-1160	LQPELDSFKEELDKYFKNHT
P-S116	1151-1170	ELDKYFKNHTSPDVDLGDIS
P-S118	1171-1190	GINASVVNIQKEIDRLNEVA
P-S119	1181-1200	KEIDRLNEVAKNLNESLIDL
P-S120	1191-1210	KNLNESLIDLQELGKYEQYI
P-S121	1201-1220	QELGKYEQYIKWPWYIWLGF
P-S127	1261-1273	SEPVLKGVKLHYT
P-E6	51-70	LVKPSFYVVYSRVKNLNSSRV
P-E7	61-75	RVKNLNSSRVPDLLV
P-M1	1-20	MADSNGTITVEELKKLLEQW
P-M2	11-30	EELKKLLEQWNLVIGFLFLT
P-M4	31-50	WICLLQFAYANRNRFLYIIK
P-M11	101-120	RLFARTRSMWSFNPETNILL
P-M12	111-130	SFNPETNILLNVPLHGTILT
P-M14	131-150	RPLLESELVIGAVILRGHLR
P-M15	141-160	GAVILRGHLRIAGHHLGRCD
P-M16	151-170	IAGHHLGRCDIKDLPKEITV
P-M17	161-180	IKDLPKEITVATSRTLSYYK
P-M18	171-190	ATSRTLSYYKLGASQRVAGD
P-M19	181-200	LGASQRVAGDSGFAAYSRYR
P-M20	191-210	SGFAAYSRYRIGNYKLNTDH
P-M21	201-220	IGNYKLNTDHSSSSDNIALL
P-N2	11-30	NAPRITFGGPSDSTGSNQNG
P-N3	21-40	SDSTGSNQNGERSGARSKQR
P-N5	41-60	RPQGLPNNTASWFTALTQHG
P-N6	51-70	SWFTALTQHGKEDLKFPRGQ

P-N7	61-80	KEDLKFPRGQGVPINTNSSP
P-N8	71-90	GVPINTNSSPDDQIGYYRRA
P-N10	91-110	TRRIRGGDGKMKDLSPRWYF
P-N11	101-120	MKDLSPRWYFYYLGTGPEAG
P-N12	111-130	YYLGTGPEAGLPYGANKDGI
P-N13	121-140	LPYGANKDGIIWVATEGALN
P-N14	131-150	IWVATEGALNTPKDHIGTRN
P-N15	141-160	TPKDHIGTRNPANNAAIVLQ
P-N16	151-170	PANNAAIVLQLPQGTTLPKG
P-N17	161-180	LPQGTTLPKGFYAEGSRGGS
P-N19	181-200	QASSRSSSRSRNSSRNSTPG
P-N20	191-210	RNSSRNSTPGSSRGTSPARM
P-N21	201-220	SSRGTSPARMAGNGGDAALA
P-N22	211-230	AGNGGDAALALLLLDRLNQL
P-N23	221-240	LLLLDRLNQLESKMSGKGQQ
P-N24	231-250	ESKMSGKGQQQQGQTVTKKS
P-N25	241-260	QQGQTVTKKSAAEASKKPRQ
P-N26	251-270	AAEASKKPRQKRTATKAYNV
P-N27	261-280	KRTATKAYNVTQAFGRRGPE
P-N28	271-290	TQAFGRRGPEQTQGNFGDQE
P-N29	281-300	QTQGNFGDQELIRQGTDYKH
P-N30	291-310	LIRQGTDYKHWPQIAQFAPS
P-N31	301-320	WPQIAQFAPSASAFFGMSRI
P-N34	331-350	LTYTGAIKLDDKDPNFKDQV
P-N35	341-360	DKDPNFKDQVILLNKHIDAY
P-N36	351-370	ILLNKHIDAYKTFPPTEPKK
P-N37	361-380	KTFPPTEPKKDKKKKADETQ
P-N38	371-390	DKKKKADETQALPQRQKKQQ
P-N39	381-400	ALPQRQKKQQTVTLLPAADL
P-N40	391-410	TVTLLPAADLDDFSKQLQQS
P-N41	401-419	DDFSKQLQQSMSSADSTQA
